# The Synthetic Myeloperoxidase Inhibitor AZD3241 Ameliorates Dextran Sodium Sulfate Stimulated Experimental Colitis

**DOI:** 10.3389/fphar.2020.556020

**Published:** 2020-09-15

**Authors:** Gulfam Ahmad, Belal Chami, Yuyang Liu, Angie L. Schroder, Patrick T. San Gabriel, Antony Gao, Genevieve Fong, XiaoSuo Wang, Paul K. Witting

**Affiliations:** ^1^ Discipline of Pathology, Faculty of Medicine and Health, Charles Perkins Centre, The University of Sydney, Sydney, NSW, Australia; ^2^ Discipline of Oral Pathology, Faculty of Medicine and Health, School of Dentistry, The University of Sydney, Sydney, NSW, Australia

**Keywords:** inflammatory bowel disease, neutrophil-myeloperoxidase, host tissue damage, pharmacologic inhibitor, dextran sodium sulfate

## Abstract

Chronic inflammatory bowel disease (IBD) is a condition with multifactorial pathophysiology. To date, there is no permanent cure and the disease is primarily managed by immunosuppressive drugs; long-term use promotes serious side effects including increased risk malignancies. The current study aimed to target neutrophil-myeloperoxidase, a key contributor to the pathogenesis of IBD, through the use of AZD3241that inhibits extracellular myeloperoxidase. Experimental colitis was induced in C57BL/6 male mice by 2% dextran sodium sulfate in drinking water *ad libitum* over 9 days. Mice received either normal drinking water and peanut butter (control), 2% DSS in drinking water and peanut butter or 2% DSS in drinking water and AZD3241 (30 mg/kg) dispersed in peanut butter daily for 9 days. Administered AZD3241 attenuated body weight loss (10% *p<*0.05) and improved clinical score (9 fold *p<*0.05; a score comprising the time-dependent assessment of stool consistency and extent of rectal bleeding), loss of colonic crypts (*p<*0.001), preserved surface epithelium (*p<*0.001) and enhanced expression of the transcription factor Nrf-2 (regulator of antioxidants) and enhanced expression of the downstream antioxidant response element haeoxygenase-1 (HO-1) in the colon tissue. Also, the concentration of fecal hemoglobin and the myeloperoxidase specific oxidative damage biomarker 3-chlorotyrosine in the colon were significantly decreased in the presence of AZD3241. This latter result was consistent with AZD3241 inhibiting MPO activity *in vitro*. Overall, AZD3241 ameliorated the course and severity of experimental colitis through ameliorating MPO derived tissue damage and could be considered a potential therapeutic option, subject to further validation in chronic IBD models.

## Introduction

Inflammatory bowel diseases (IBD; notably Crohn’s disease and ulcerative colitis) are a debilitating group of inflammatory conditions; affected patients experience abdominal pain, vomiting, diarrhea, fever, rectal bleeding and weight loss (de Lange and Barrett, 2015). Though rarely fatal, the quality of normal life can be severely compromised and in the longer term increased risk of colorectal cancer (ranging 0.5%–20%/year) is a serious clinical complication of untreated IBD (May et al., 2011). Presently, there is no permanent cure for this group of diseases and the symptoms are managed through immunosuppressant, non-steroidal antiinflammatory (NSAID) drugs, and dietary changes to minimize environmental triggers, with severe cases resulting in surgical resection of the damaged bowel.

Current clinically prescribed IBD drugs such as sulfasalazine, corticosteroids and immunosuppressants focus on targeting the inhibition of the chemokine tumor necrosis factor (TNF) (Moura et al., 2015) and its associated downstream inflammatory response. However, the prolonged use of these drugs is associated with side effects and intermittent relapse of the disease and associated symptomatology, which limits the widespread prescription and protracted use of these drugs (Barnes and Adcock, 2009). Other pharmacological factors also complicate the use of these first line therapeutics; for example, antibiotics if given for a prolonged time alter the internal gut microbiota, which may result in drug resistance (Sartor, 2004) leading to requirement of higher effective dosing. Furthermore, sulfasalazine has been shown to sporadically aggravate colitis and induce both diarrhea and abdominal pain in some cases of IBD, indicating this drug is not universally tolerated (Tremblay et al., 2011). Overall, the severe side effects of contemporary treatments combined with relatively high out-of-pocket costs of these drugs either taken as mono or combinatorial therapies justifies investigations aiming to develop alternative therapeutic approaches precisely targeting the pathophysiology of the disease, which currently remains an unmet challenge in the field of gastroenterology (Fok et al., 2012).

Central to pathophysiology of IBD is the active recruitment of neutrophils (and to a lesser extent monocytes) which rapidly infiltrate at the site of injury and are considered the first line of defense against invading pathogens (Kaplanski et al., 2003; Chami et al., 2018) albeit that these cells also cause host tissue damage. These immune cells, primarily the neutrophils, degranulate to release the homodimeric heme enzyme myeloperoxidase (MPO), which performs its bactericidal function *via* the halogenation cycle by producing a variety of hypohalous acids including the potent antibactericidal oxidant hypochlorous acid (HOCl) in the presence of halides and hydrogen peroxide (Chami et al., 2018).

Notably plasma MPO is elevated in IBD patients and fecal MPO shows positive correlation with IBD disease severity (Saiki, 1998; Sangfelt et al., 2001; Peterson et al., 2002; Wagner et al., 2008). The potent oxidant HOCl is the primary antibactericide produced by neutrophil-MPO and this two-electron oxidant readily diffuses and reacts with a vast array of biological targets including tyrosine residues to form 3-chlorotyrosine (3-Cl-Tyr), which represents as a surrogate marker for MPO-activity *in vivo*. Unlike other biomarkers of oxidative stress, 3-Cl-Tyr is heat stable, can be extracted from tissues following degradative protein pyrolysis and represents an ideal measure of MPO-derived oxidative tissue damage (Chami et al., 2018). Consistent with MPO potentially playing a role in the pathogenesis of IBD, inflamed mouse colon and colon specimens from patients with both ulcerative colitis (UC) and Crohn’s disease (CD) show higher 3-Cl-Tyr and MPO levels than corresponding healthy control tissue (Knutson et al., 2013).

As indicated above, neutrophils are the major producers of MPO which generate HOCl that eventually leads to more tissue damage. This cascade tends to increase chemotactic response by eliciting affected cells to produce chemokines such as interleukin-18 (IL-18) (Jucaite et al., 2015), thereby recruiting more neutrophils and consequently resulting in a cycle of aggravated inflammatory response - a distinctive hallmark of IBD. Therefore, targeting MPO activity/inhibition over the short-term could potentially reduce the acute inflammatory response and also diminish the severity of disease. Intermittent targeting of MPO may offer an alternative (or adjunctive) approach to the raft of current immunosuppressive IBD treatment options; longer term MPO inhibition may impact on the adaptive response and have consequences on the host-defense against pathogenic microorganisms. The current study aims to test the potential therapeutic activity of the irreversible MPO inhibitor (AZD3241 also known as BHV-3241; Biohaven Pharmaceuticals) in an experimental mouse model of acute colitis stimulated by consuming 2% w/v dextran sodium sulfate (DSS) in drinking water.

## Methods

### Animals

All experiments were conducted in line with protocols approved by the Animal Ethics Committee (approval #1496), University of Sydney. Male C57BL/6 mice of 6 weeks age were purchased from Animal Resources Centre, Perth, Australia and housed at Laboratory Animal Services (Charles Perkins Centre, Sydney University, Australia) in environmentally enriched husbandry cages. Mice were acclimated for one week under 12 h day and light cycle at 22°C and fed on normal chow and tap water *ad libitum* for the duration for the study. Prior to study commencement each mouse was ear-tagged for individual identification purposes over the course of the treatment.

### Experimental Groups

After acclimation, animals were randomly divided into three experimental groups consisting of five animals per group. Each group of mice was housed in a single humidified cage as follows:

- Control/vehicle group receiving *water ad libitum* for 9 days
- DSS group receiving 2% w/v DSS in drinking *water ad libitum* for 9 days
- DSS+AZD3241 group receiving 2% w/v DSS in drinking *water ad libitum* and AZD3241 administered orally @30mg/kg body weight dispersed into peanut butter (~0.1 g) daily for 9 days

Commonly 3%–5% DSS is employed to develop experimental IBD over 5–7 days; this yields a highly aggressive acute disease characterized by acute weight loss stemming from an inability to seek food and water due to severe diarrhea that also limits the optimal absorption of orally available drugs/treatment. Here we employed 2% DSS to induce a milder onset of disease over 9 days that yields classical clinical symptoms of diarrhea, weight loss and rectal bleeding.

The dose of AZD3241 was suggested after consultation with the Drs Wolfgang Jarolimek and Jonathan Foot (Pharmaxis Ltd, Frenchs Forest, Sydney, Australia) and was predicated on phase 2 human clinical trials where 1.2 g was administered daily for 8-12 weeks (Jucaite et al., 2015; Tong et al., 2018). Based on our previous experience with DSS induced colitis, animals consistently start losing weight from day 7 onwards (with slight variation in individual animal response) when provided 2% w/v DSS in the drinking water and achieve a maximum 15% weight loss threshold (relative to weight of the individual mouse measured at day 0) as set by local animal ethics committee commonly occurring between days 8-9 of DSS insult.

### *In Vitro* Assessment of MPO Inhibition by AZD3241

The efficacy of AZD3241 to inhibit MPO activity was initially tested using an *in vitro* luminescent assay developed in our lab as previously described (Chami et al., 2020). Briefly, a solution comprising 80 μl each of 0.2 µg/ml MPO and 0.8 mM (w/v) luminol was added to the wells of a commercial 96 well plate (Corning-Life Sciences, Australia). Next, each well was supplemented with 18 µl of NaCl 150 mM (w/v) and 20 µl AZD3241 (supplied by Pharmaxis, Frenchs Forest, Sydney, Australia) at four different final concentrations (0, 1.35, 6.75, and 13.5 μM). Following treatment, the reaction plate was agitated slowly on an orbital shaker (150 r.p.m., 15 min, 22°C). Next, the peroxidase reaction was initiated by addition of H_2_O_2_ (2 μl; 45 mM (v/v)); this peroxide was carefully added to the side of each well and just before reading, the plate was gently tapped to allow the contents to fall and mix with the other constituents of the reaction mixture. The evolved luminescence signal was immediately measured using an IVIS^®^ SpectrumCT In Vivo Imaging System (PerkinElmer, Australia) and monitoring continued every 30 s for a total of 10 min to generate a reaction velocity curve. Finally, the well luminescence was normalized to total photo flux per well to obtain a total luminescent output for each of the treatments that was subsequently averaged for each grouping.

### Oral Drug Administration

The MPO inhibitor (AZD3241) was supplied by our industry partner Pharmaxis Ltd, NSW Australia (purity ~96% as assessed by 1H NMR, data not shown). Before commencement of experiments, mice were routinely trained to accept peanut butter *via* oral intake to mimic currently prescribed orally administered IBD drugs in humans and AZD3241 intake in phase 1 and 2 clinical trials (Jucaite et al., 2015; Tong et al., 2018). After 12 h overnight fasting, mice from an allocated group were briefly rehoused individually in cages and provided with peanut butter (50–100 mg) presented on small pieces of plastic cut from a weighing boat that was temporarily adhered to the floor of the cage using “Bostic Blu Tak”^®^ removable adhesive. Animals were continuously monitored until they consumed the entire aliquot of peanut butter. In 3–5 days, animals were suitably trained, with the time taken to consume the peanut butter averaging 30 min after provision of this carrier.

After all the mice in the designated experimental groups had been adequately trained to consume peanut butter, the experimental protocol was initiated with AZD3241 provided @ 30 mg/kg body weight mixed into peanut butter as follows: vehicle (control) and DSS (alone) groups received peanut butter with no added drug, while mice designated to the drug treatment group received AZD3241 daily in conjunction with DSS insult that continued over the duration of monitoring. Throughout the monitoring period animals from all groups continued to consume the daily dose of peanut butter. However, it was noted that animals assigned to the DSS+AZD3241 group required a relatively longer time to completely consume the drug-treated peanut butter.

### Macroscopic Evaluation of Experimental Colitis

As a consequence of the relatively acute nature of disease development in this model, disease progression and severity were monitored and recorded daily after commencement of treatment through the assessment of four gross observational measures, enumerated to give a combined clinical scoring as we and others have described earlier (Kim et al., 2012; Chami et al., 2020).

1. Body Weight: parameters: (a) No weight loss = 0; (b) 1%–10% loss = 1; (c) >10% loss = 2
2. Stool and/or bleeding: parameters: (a) Normal consistency of stool = 0; (b) Soft stool = 1; (c) Watery/bloody stool = 2
3. Rectal prolapse: parameters: (a) No prolapse = 0; (b) Prolapse present = 1
4. Grooming: parameters: (a) No hunched posture, bristle fur, or skin lesions = 0; (b) Presence of hunched posture, bristle fur and skin lesions = 1

The combined score for each mouse displaying these criteria in each group was then averaged across the entire group to ascertain the impact of DSS +/- intervention.

### Real-Time Monitoring of Intestinal Inflammation

On day 9 before culling, animals were imaged to assess bioluminescence using imaging equipment housed in the preclinical imaging facility at Charles Perkins Centre at Sydney University. Upon systemic administration, luminol generates a bioluminescence signal that can be imaged through the non-specific oxidation of luminol by peroxidase activity/reactive species such as superoxide radical anion, peroxynitrite and H_2_O_2_ to yield a chemiluminescent product (Tarpey and Fridovich, 2001). Briefly, a stock solution of disodium 5-amino-2,3-dihydro-1,4-phthalazine-dione (luminol, final concentration 475 mM) was prepared in phosphate buffered saline (pH 7.2). Where required, 100 µl of this stock luminol solution was injected into each mouse *via* a subcutaneous (SC) route under anesthesia (1.5%–2% v/v isoflurane) followed by imaging using the IVIS^®^ SpectrumCT (PerkinElmer) with a 3 min exposure time (F/stop = 1; binning = 1). Luminol possesses redox-sensitive properties and emits blue luminescence (λ_max_ = 425 nm) after reacting with MPO-derived HOCl (Gross et al., 2009).

### Urine and Fecal Sample Collection

Immediately following completion of *in vivo* bioluminescent imaging, animals were re-anesthetized under 5% v/v isoflurane before euthanised *via* cervical dislocation. Urine was collected either by neck scuffing before initiating deep anesthesia or taken directly from the bladder (using a 1 ml syringe fitted with a 25 G needle, Terumo, Australia) during the surgical resection of organs from each mouse. To avoid external contamination, fecal samples were collected directly from the resected colons by gently squeezing out the fecal material into the collection tube. Samples were snap frozen in liquid nitrogen and transferred to −80°C for storage until analyzed for the presence of hemoglobin.

Upon sacrifice, colons were immediately excised between the anus to the ascending colon. Isolated colon specimens were flushed twice with cold 10 mM phosphate buffered saline (PBS) using a syringe and then evenly divided into two longitudinal pieces with a surgical blade and prepared separately for histological and biochemical analysis.

### Colon Embedding, Sectioning, and Homogenization

One-half of the longitudinal colon strip was horizontally cut into four pieces and placed into separate cassettes. Cassettes containing tissue samples were then immersed in 100% ethanol overnight and transferred to an automated tissue processor for fixation. Following this, samples were embedded into paraffin and stored at 22 °C. Paraffin sections (thickness 5 μm) were produced using a rotary microtome and collected onto glass slides, which were then dried for 1 h at 60°C and later stained with hematoxylin and eosin for histological/tissue architectural examination.

The remaining portion of colon samples and all fecal samples collected were prepared for subsequent homogenization. Briefly, samples were snap frozen in liquid nitrogen, grounded into a fine powder using a mortar and pestle, transferred into a glass vial and then treated with 1 ml complete lysis buffer containing: phosphate buffer (50 mM, pH 7.4), 1 protease inhibitor cocktail tablet (Roche)?, 1 mM ethylenediaminetetraacetic acid (EDTA), 10 µM butylated hydroxytoluene (BHT). The mixture was then homogenized using a rotating piston and matching Teflon-coated tube (Wheaton Scientific (now DWK Life Sciences) Millville, NJ, USA) operating at 500 revolutions per minute (r.p.m). After 5 min, homogenized tissues were transferred into two prelabeled capped-tubes and centrifuged (4°C, 12,000 r.p.m.) for 15 min. The supernatant was collected, and protein concentration was assessed using the bicinchoninic acid (BCA) as per manufacturer’s instructions (add manufacturer). Finally, the clarified homogenates were aliquoted and stored in −80°C for further biochemical analyses.

### Fecal Hb and Calprotectin Analysis

Homogenized fecal supernatants were thawed at 22°C and then an aliquot (200 µl) of each sample was added into a 96-well plate. The content of fecal Hb was then measured in the Tecan M200 Pro at 405 nm wavelength; this wavelength was selected as it was close to the Soret maximum for ferric Hb. In other analyses, fecal calprotectin levels were measured by ELISA as per manufacturer’s instructions (Jomar Life Science, Australia) and concentration of the analyte calculated by comparison with authentic standards.

### Assessment of MPO Enzyme Activity in the Colon

Assessment of 3-Chloro-tyrosine (3-Chlor-Tyr) a biomarker of MPO activity was determined as previously published (Chami et al., 2020). Briefly, colon tissue homogenates were delipidated and precipitated by the addition of 25 µl of 0.3% w/v deoxycholic acid and 50 µl of 50% w/v trichloroacetic acid (TCA) over incubation for 5 min. Vials were then transferred into 2 ml centrifuge tubes and centrifuged (2 min, 5°C, 9,000 r.p.m). The supernatants were discarded and the pellets of protein precipitate were washed with 5% w/v TCA (100 µl) and incubated for 3 min before being centrifuged (2 min, 5°C, 9,000 r.p.m). The resultant supernatant was removed, and the process was repeated for two more rounds of centrifugation with the addition of 100 µl ice-cold 100% acetone instead of 5% w/v TCA. Next, the isolated pellets were then left to airdry in a fume hood for 30 min. During this time, an internal standard mixture was prepared by mixing 1 µM labeled tyrosine and 1 µM labeled 3-Chlor-Tyr in 1:1 v/v ratio with intermittent mixing by vortex.

Following this processing, each vial containing a dry protein pellet was treated with 150 µl of 4M methanesulfonic acid containing 0.2% w/v tyramine [Sigma Aldrich, Australia (M4141)] and 10 µl of internal standard mixture. Notably, HCl was not used in this acidification step to avoid the possibility of chlorination reactions in the presence of this hard acid. Next, forceps were used to transfer glass vials into a hydrolysis reaction vial [PicoTag Reaction vials; Eldex laboratories, USA (1163)], with six vials fitted into one pyrolysis vessel. Following this process, the vessels were degassed by connecting a vacuum pump for 2 min and then the vial was back flushed for 2 min with Argon to thoroughly degas the sample. This process was repeated 6× and finalized with a 2-min vacuum pump evacuation before the vessels were transferred to an oven (Labwit Scientific, ZXRD-A5055, Vic. Australia)) and heated to 110°C for 16 h to promote acid-pyrolysis of the bowel specimens.

Next, each tissue specimen was purified for mass spectrometry by using solid phase extraction purification of hydrolysates with absolute methanol-activated columns and prior preconditioning with 0.1% v/v TFA/H_2_O. Samples were loaded onto each column and columns were washed thoroughly with formic acid (0.1% v/v, 2 ml) and 3-Chlor-Tyr eluted with 80% v/v methanol/H_2_O. The eluted samples were air dried under vacuum at 60°C and the resultant solid residue re-dissolved in formic acid (0.1% v/v 100 μl). The samples were then submitted to a Shimadzu triple quadrupole LCMS system (LCMS-8050, Shimadzu Co., Kyoto, Japan) for MRM (multiple reaction monitoring) analysis on each analyte. Ion chromatographic peaks for each analyte [*e.g.*, tyrosine, labeled tyrosine, 3-Cl-Tyrosine and labeled 3-Cl-Tyrosine] were identified by comparison to the corresponding authentic standard, integrated and calculated using LabSolution**^©^** software system (Shimadzu Corporation, Japan, V 5.89). Analyte concentrations were calculated based on the corresponding standard curve and presented as the ratio relative to their corresponding isotopically labeled form measured in the same sample.

### Colon Histomorphology and Cellular Infiltration Examination

Thin longitudinal sections were stained with hematoxylin and eosin and examined under light microscopy (Zeiss Axioscope, Talavera Rd, Sydney, Australia) for pathological scoring and cellular infiltration (neutrophils) by an experienced pathologist. Multiple sections of 1–2 cm colon/animal were scanned and scored using the pathological criteria defined in **Table 1**.

**Table 1:** Colon histomorphology and neutrophil infiltration scoring criteria.

Category	Histological criterion	Score value
Crypts	Intact crypts	0
	Disoriented crypts	1
	Variable crypt diameter	2
	Atrophied crypts	3
	Mucosa devoid of crypts	4
Loss of surface epithelium	Intact surface epithelium	0
	Sloughing off epithelial surface	1
	Patchy loss of surface epithelium	2
	Moderate loss of surface epithelium	3
	Severe loss/erosion of surface epithelium	4
Cellular infiltration	Clear mucosa/submucosa	0
	Mucosal/lamina propria infiltration	1
	Mucosal and submucosal infiltration	2
	Moderate cryptitis/infiltration to crypt epithelial cells	3
	Severe cryptitis	4

### Immunofluorescence Studies

Expression of nuclear factor (erythroid-derived 2) (NF-E2)-related factor 2 (Nrf2), a key regulator of antioxidant response genes such and hemoxygensae-1 (HO-1) were studied by immunofluorescence staining. Paraffin embedded colon specimens were sectioned at five microns and placed on frosted glass slides that were baked in an oven set to 67°C for 1 h to allow sufficient adherence of the tissue onto the slide. Heat-treated slides were then dewaxed with xylene (2 × solvent changes,10 min/change) and rehydrated in graded alcohol (100, 80, 70% v/v), incubating the slides for 2 min at each alcohol concentration. Antigen retrieval (heat retrieval) was performed in a Biocare Decloaking Chamber (DAKO Cytomation, CA) for 40 min.

Next, slides were incubated in 3% v/v methanolic H_2_O_2_ and Dako protein block (serum free) for 30 min in order to block endogenous peroxidase activity and non-specific immunoreactivity respectively. Specimens were incubated together with primary Nrf2 and HO-1 antibodies (1:200 and 1:500 v/v respectively) for 1 h followed by envision or secondary antibody incubation for 30 min at 22 °C. After 10 min of incubation in Opal fluorophore, specimens were incubated in 0.5% w/v Sudan Black B for 10 min to quench any autofluorescence followed by DAPI staining for 5 min. Slides were analyzed under an upright fluorescent microscope (ZEISS AXIOscope; Talavera Rd, Sydney, Australia).

### Statistical Analysis

Where relevant data are presented as mean±SD and analyzed using GraphPad Prism^®^ (*v*8) software by applying ordinary one-way Analysis of Variance (ANOVA) with Tukey post-hoc test for multiple comparisons. Significance was accepted at the 95% confidence interval and represented as *p<*0.05.

## Results

### AZD3241 Mitigates HOCl-Mediated Oxidation of Luminol *In Vitro*

**Figure 1:**
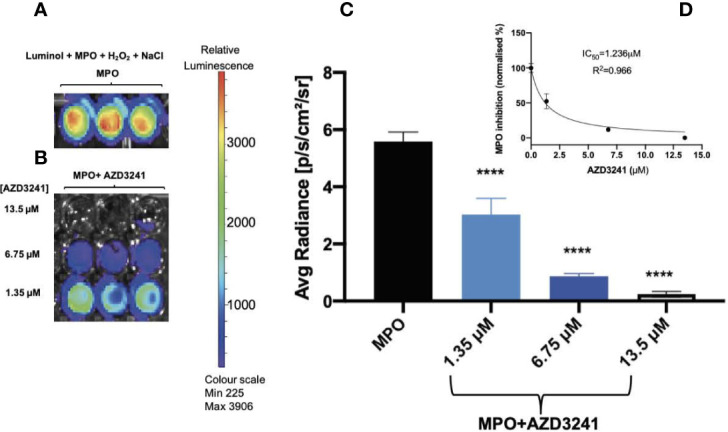
AZD3241 inhibits myeloperoxidase (MPO)/hypochlorous acid (HOCl)-mediated oxidation of luminol *in vitro*. **(A)** MPO/HOCl-mediated oxidation of luminol in the presence of NaCl (150 mM). **(B)** Representative **(C)** dose dependent quantitative analysis of AZD3241 inhibition on MPO activity supplemented with NaCl and **(D)** Estimated IC_50_ of AZD3241 from curve fitting performed using GraphPad Prism software (*v*8). Right side vertical scale indicates the relative luminescence radiance shown as a heat map with relative radiance scale. Data in **(C, D)** represent mean ± SD quantification of three independent replicates measured in units of photons/sec/cm^2^/sr. ^****^Different to MPO control group (*p <* 0.0001). On all figures “AZD3241” is abbreviated as “AZD”.

Isolated human MPO is a peroxidase that can produce HOCl in the presence of H_2_O_2_ and excess chloride ions (Cl^-^). An intense luminescence signal is produced when luminol, a substrate for MPO peroxidase, undergoes MPO-mediated oxidation. Our *in vitro* results indicate a significant MPO-derived luminol oxidation in the presence of NaCl (but in the absence of drug AZD3241) as indicated by a strong luminescence signal with mean radiance greater than 3500 as shown using a heat map readout that reflected relative luminescence (**Figure 1A**). In contrast, supplementation with AZD3241 applied to the reaction mixtures at various concentrations (0, 1.35, 6.75 and 13.5 μM) showed a dose-dependent reduction in the extent of luminol oxidation in the presence of human MPO (**Figures 1B, C**) indicating that the drug was able to effectively mitigate MPO enzymic activity as detected by monitoring luminol oxidation in this *in vitro* setting. Using this data, the IC_50_ was estimated to be 1.2 μM (refer to inset to **Figure 1D**); based on this positive outcome from *in vitro* assessment of the inhibitory action of AZD3241 in the MPO/HOCl enzymic activity assay, we proceeded to conduct *in vivo* experiments to assess the capacity of the pharmacological inhibitor in ameliorating MPO activity in an experimental murine model of colon inflammation.

### AZD3241 Improves Clinical Outcomes of DSS Induced Colitis

#### Body Weight Loss

**Figure 2:**
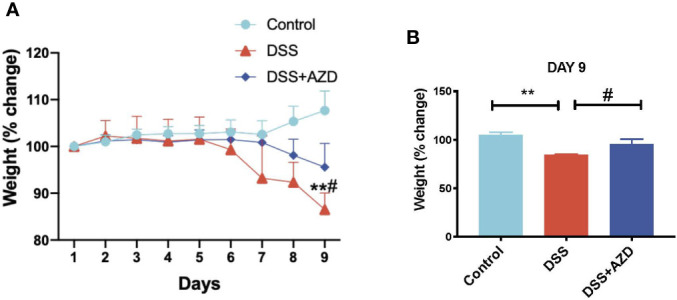
Orally available AZD3241 attenuates body weight loss in mice challenged with dextran sodium sulfate (DSS). Mice were randomly divided into three treatment arms over 9 days i) vehicle group (n=5) given normal drinking water and peanut butter without drug; ii) DSS group (n=5) given 2% w/v DSS in drinking water and peanut butter without drug; iii) DSS+AZD3241 (n=5) given 2% w/v DSS in drinking water and AZD3241 @ 30 mg/kg in peanut butter. **(A)** Percent body weight loss in mice over 9 days. **(B)** Percent body weight loss at day 9 of experiments. **Different to the control, (*p < *0.001). ^#^Different to the DSS+AZD3241 group, (p = 0.01). AZD3241 is abbreviated to AZD in this figure.

Mice were weighed daily for 9 days and loss/gain in weight was compared the body weight at day 0 of challenge. It was noted that a marked decline in animal weight was observed in the absence of drug treatment as early as day 6 of DSS challenge, which reached a significant 15% weight loss (*p<*0.05) at day 9 relative to the corresponding average weight on day 1 for the same cohort. By contrast, mice receiving AZD3241 showed resistance to weight loss at day 9 with only a minor (5%) decline in weight recorded at day 9 relative to the corresponding average weight on day 1 for the same cohort (**Figures 2A** and inset B). These data indicate that AZD3241 preserved the general health of mice and their corresponding weight was maintained for a longer time than determined in mice supplemented DSS alone in the drinking water. In addition, mice supplemented AZD3241 showed resistance to colitis development, which consequentially resulted in relatively normal food seeking and consumption behavior, regulating maintenance of intestinal absorption and an overall preservation of body weight.

#### Stool Consistency, Rectal Prolapse, and General Animal Health Score

**Figure 3:**
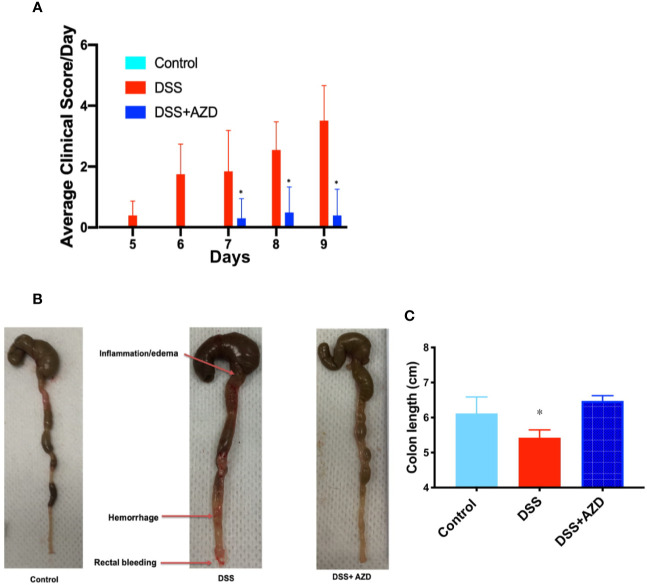
**(A)** Enumerated clinical score representing fecal consistency, rectal bleeding, reduced mobility and general health of the animals over 9 days of treatment. **(B)** Representative images of colon morphology resected after 9 days. **(C)** Mean colon length at day 9. Mice were randomly divided into three treatment arms over 9 days i) vehicle group (n=5) given normal drinking water and peanut butter without drug; ii) dextran sodium sulfate (DSS) group (n=5) given 2% w/v DSS in drinking water and peanut butter without drug; iii) DSS+AZD3241 (n=5) given 2% w/v DSS in drinking water and AZD3241 @ 30 mg/kg in peanut butter. Different to the DSS group, (*p = 0.001). AZD3241 is abbreviated to AZD in this figure.

Next, a combined clinical score was assigned among the experimental groups using gross observational criteria as indicated in methods section. Overall, mice designated to the DSS-insult group increasingly displayed loose stool at day 5, which worsened on each day of monitoring until the mice displayed evidence of rectal bleeding/prolapse and compromised general health at day 9. By contrast, mice designated to the DSS+AZD3241 group showed a delayed set of symptoms with loose stool only detected at day 7 and minimal blood spotting in feces (identified as rectal bleeding) compared to DSS group at day 9. Overall clinical score for mice in the DSS+AZD3241 group was significantly improved when compared to mice receiving DSS alone (**Figure 3A**) although, this did not reach the level of the corresponding control group.

Resected colons from all experimental groups were compared for gross morphological examination (**Figure 3B**) and colon length (**Figure 3C**). Mice that were supplemented DSS alone in the drinking water showed regions of the colon that were clearly inflamed, oedematous and in some regions hemorrhagic, which manifested as reduced colon length. By contrast, the colons obtained from mice supplemented both DSS+AZD3241 showed preserved gross colon morphology and length with an absence of hemorrhage and inflammation; overall macroscopic appearance was comparable to colon from control mice (**Figure 3B**). These data align completely with the improved clinical scores recorded for the drug-supplemented group.

### AZD3241 Mitigates MPO/HOCl Mediated *In Vivo* Bioluminescence

**Figure 4:**
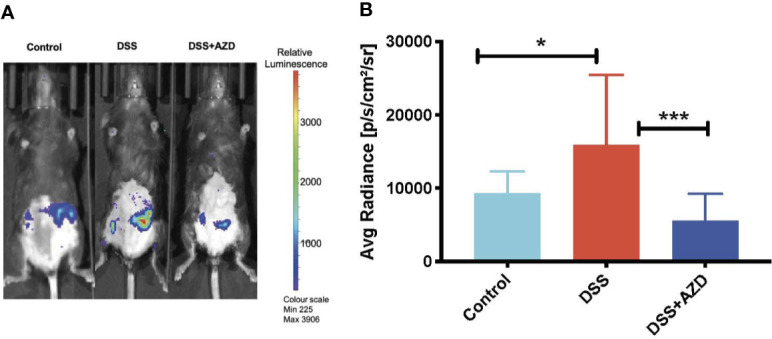
*In vivo* bioluminescent imaging of luminol oxidation in mice. Mice were randomly divided into three treatment arms over 9 days i) vehicle group given normal drinking water and peanut butter without drug; ii) dextran sodium sulfate (DSS) group given 2% w/v DSS in drinking water and peanut butter without drug; iii) DSS+AZD3241 given 2% w/v DSS in drinking water and AZD3241 @ 30 mg/kg in peanut butter. At day 10, mice were anesthetized, maintained at 1.5%–2% v/v isoflurane and injected with 100 µl of 475 mM luminol through subcutaneous (SC) route in the scruff and immediately imaged using IVIS^®^ SpectrumCT (PerkinElmer). **(A)** Representative *in vivo* images of bioluminescence signal in vehicle control (left), 2% w/v DSS (middle) and 2% w/v DSS+AZD3241 right. **(B)** Quantification of regions of interests (ROIs) depicting highest bioluminescence signal intensity. Right side vertical scale is indicative of relative bioluminescence radiance shown as a heat map with relative radiance scale. Data is representative of n=4/group presented as radiance measured in units photons/sec/cm^2^/sr. *Different to the control, (p = 0.01); ***Different to the DSS+AZD3241 (*p <* 0.001). AZD3241 is abbreviated to AZD in this figure.

To investigate the MPO-inhibitory activity of AZD3241 *in vivo*, luminol (100 µl, 475 mM) was administered subcutaneously (SC) and mice were immediately imaged under anesthesia using the IVIS^®^ SpectrumCT instrument as described in methods section. As indicated by our *in vitro* studies (**Figure 1**) MPO-generated HOCl is capable of rapidly oxidizing luminol that is measured by the emitted luminescence. Thus, bioluminescence was captured immediately after injecting the mice with luminol. Quantification of this *in situ* signal indicated that mice treated with DSS alone stimulated a marked increase in bioluminescence that was localized to the inflamed colon. The mean MPO signal intensity in DSS inflamed colon was 1.7 fold (~70%) higher than the controls in the absence of DSS insult. In mice co-supplemented DSS+AZD3241 the corresponding abdominal bioluminescent signal was significantly lower compared to that measured in the colons of mice receiving DSS alone (~3 fold) suggesting that the drug was able to inhibit the enzymic activity of its molecular target in a complex biological environment (*c.f*., **Figures 4A, B**). The diminution of this signal in the presence of AZD3241 suggested that the supplemented drug inhibited MPO activity by ~60% when assessed using this method; overall this level of inhibition appeared to inhibit the pathogenesis in the colon. Taken together, these data indicate that neutrophils recruited to the inflamed colon release active MPO/HOCl that can drive the oxidation of luminol, which is sensitive to the irreversible MPO inhibitor AZD3241.

### AZD3241 Improves Colon Histomorphology But Has No Impact on the Extent of Cellular Infiltration

**Figure 5:**
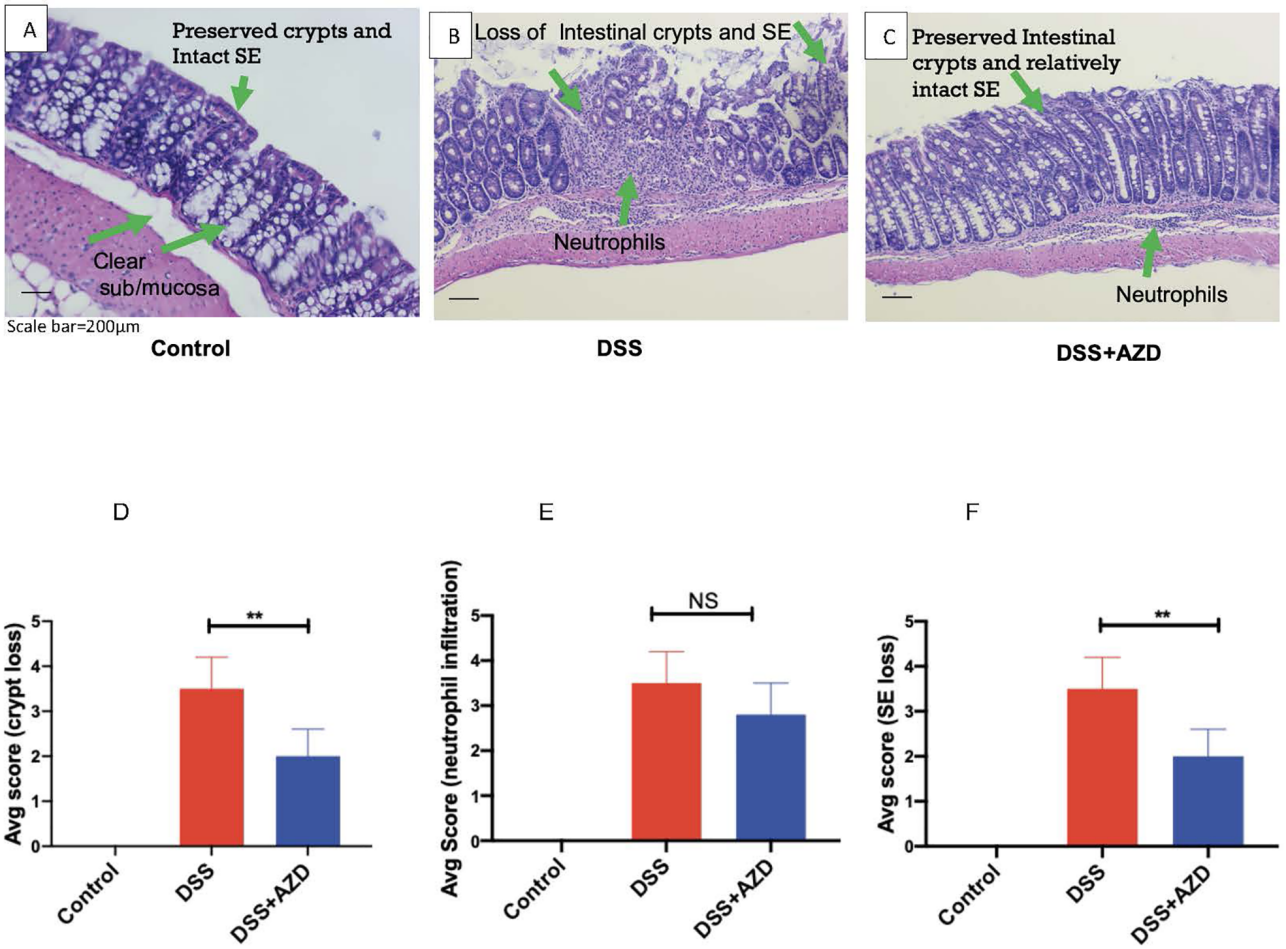
Representative histopathological images of mouse colon sacrificed and stained with Harris hematoxylin and counter stained with eosin after 9 days of dextran sodium sulfate (DSS) challenge. Mice were randomly divided into three treatment arms over 9 days i) vehicle group (n = 5) given normal drinking water and peanut butter without drug; ii) DSS group (n = 5) given 2% w/v DSS in drinking water and peanut butter without drug; iii) DSS+AZD3241 (n = 5) given 2% w/v DSS in drinking water and AZD3241 @ 30 mg/kg in peanut butter. The extent of **(A)** Crypt loss **(B)** neutrophil infiltration and **(C)** surface epithelium loss was assessed microscopically against the scoring criteria defined and in **Table 1** and is presented in **(D**–**F)**, respectively. **Different to the DSS+AZD3241, (*p < *0.001). AZD3241 is abbreviated to AZD in this figure.

Histoarchitecture of longitudinally cut thin colon sections stained with H&E was observed using light microscopy and scored for three pathological categories as described in **Table 1**. The loss of mucosal crypts was significantly greater in mice from the DSS group, which was markedly, though not completely protected, by co-supplementation of DSS+AZD3241. On closer inspection it was evident that following treatment of DSS alone, sections of the colon showed complete loss of crypts with severe erosion of the colon mucosa (**Figures 5A–D**). Colons from mice co-supplemented AZD3241 showed significant improvement in loss of colon surface epithelia, which again indicated that inhibition of MPO bioactivity may lead to retention of the gut architecture and maintenance of cell types that comprise the colon epithelium (*c.f*., **Figures 5A–C, F**).

Interestingly, despite the preservation of colon integrity in mice treated with AZD3241, the extent of cellular infiltration in the same colons was not significantly different from mice treated with DSS alone. In the latter case, the colons exhibited severe neutrophil infiltration in the submucosa and mucosa with neutrophils also present within the intercellular spaces of the crypt epithelia. A similar pattern of neutrophil infiltration was detected in mice treated with AZD3241 but with relatively less infiltrating cells detected in crypts for this group (*c.f*., **Figures 5A–C, E**). These findings indicate that AZD3241 was largely ineffective in decreasing the extent of neutrophil recruitment to the inflamed colon particularly at the sites of colitis consistent with this drug not markedly inhibiting chemotaxis. However, taken together with the outcome from the *in vivo* luminol assay, these data sets indicate that the drug was active in attenuating downstream MPO activity that manifests as decreased extent of HOCl-mediated host tissue damage.

#### AZD3241 Attenuates Fecal Markers of Neutrophil Activation and Bleeding

**Figure 6:**
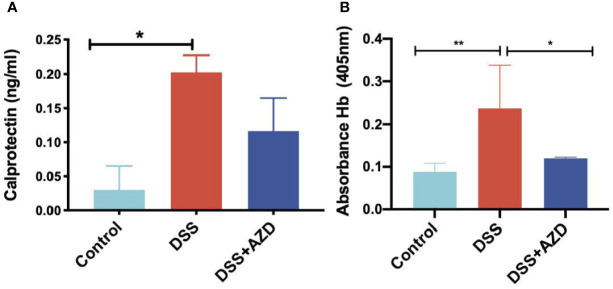
Quantification of fecal calprotectin **(A)** with (ELISA as described in methods section and fecal Hb **(B)** in fecal homogenates using Tecan M200 Pro at 405 nm wavelength. Mice were randomly divided into three treatment arms over 9 days i) vehicle group (n = 5) given normal drinking water and peanut butter without drug; ii) DSS group (n = 5) given 2% w/v dextran sodium sulfate (DSS) in drinking water and peanut butter without drug; iii) DSS+AZD3241 (n = 5) given 2% w/v DSS in drinking water and AZD3241 @ 30 mg/kg in peanut butter. Snap frozen fecal samples in liquid nitrogen were grounded into a fine powder using a mortar and pestle, transferred into a glass vial and then treated with 1 ml complete lysis buffer as described in methods section, centrifuged and supernatant separated for subsequent analyses. *Different to the control (6A), (p = 0.04); *Different to the control, (p = 0.02) and **different to DSS+AZD3241, (p = 0.005) (6B). Note, AZD3241 is abbreviated to AZD in this figure.

Fecal calprotectin levels (as an indirect measure of colon inflammation) were measured by ELISA as described in methods section. Consistent with this animal model of colitis, fecal calprotectin levels in mice treated with DSS alone increased significantly when compared to controls (**Figure 6A**). However, although calprotectin levels were discernibly lower in the feces of mice co-supplemented DSS+AZD3241, this was not decreased significantly compared to the corresponding measurement in mice insulted with DSS alone. This result is internally consistent with the drugs inability to markedly decrease the extent of infiltration (above).

Analysis of fecal hemoglobin (Hb) followed a similar pattern to calprotectin with Hb levels increased significantly in the feces of mice treated with DSS alone (when compared to the control). However, for this biomarker of bleeding, treatment with AZD3241 significantly decreased fecal Hb in mice co-supplemented DSS+AZD3241 (**Figure 6B**) albeit this was not reduced to background (control) levels. Together these findings indicate that AZD3241 attenuates experimental colitis progression by preventing mucosal damage, thereby minimizing bleeding which again aligns with the corresponding clinical score and histology where preservation of the epithelial surface was evident in the drug-treated animals.

### AZD Mitigates HOCl-Mediated Oxidative Damage

**Figure 7:**
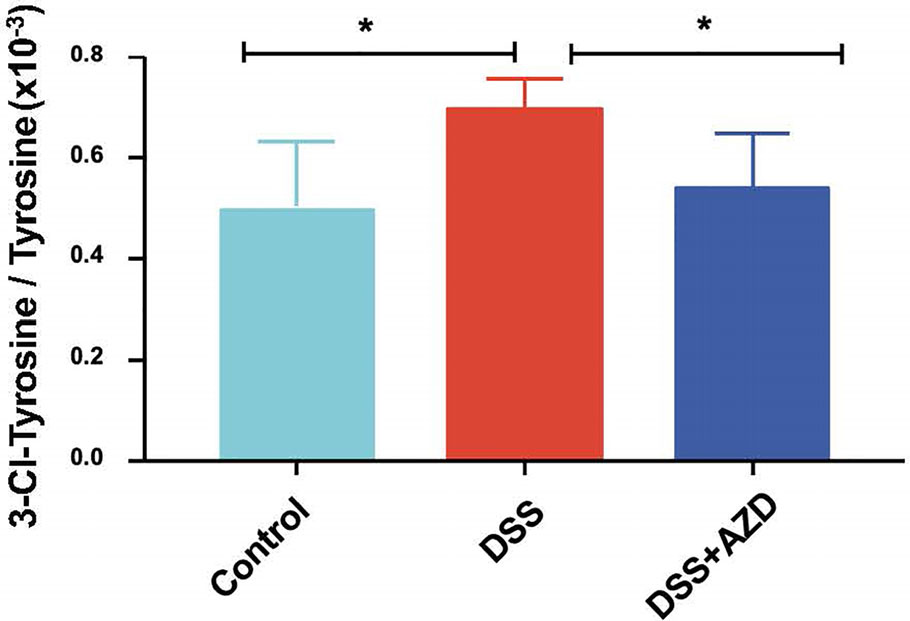
Quantification of myeloperoxidase (MPO)-mediated oxidative damage biomarker 3-Chlor-Tyr with LC mass spectrometry in colon homogenates. Mice were randomly divided into three treatment arms over 9 days i) vehicle group (n = 5) given normal drinking water and peanut butter without drug; ii) dextran sodium sulfate (DSS) group (n=5) given 2% w/v DSS in drinking water and peanut butter without drug; iii) DSS+AZD3241 (n = 5) given 2% w/v DSS in drinking water and AZD3241 @ 30 mg/kg in peanut butter. Colons were resected after 9 days of treatment, snap frozen in liquid nitrogen, grounded into a fine powder using a mortar and pestle, transferred into a glass vial and then treated with 1 ml complete lysis buffer as described in methods section, centrifuged and supernatant separated for subsequent analyses. *Different to the control and DSS+AZD3241, (p = 0.01). AZD3241 is abbreviated to AZD in this figure.

Assessment of 3-Chlor-Tyr is considered a surrogate marker of MPO activity and HOCl-mediated oxidative tissue damage; thus, levels of 3-Chlor-Tyr were determined in colon homogenates by LC/MS as described in the methods section. Treatment with AZD3241 significantly ameliorated the oxidative damage in DSS challenged mice when compared to the DSS alone group and levels of 3-Chlor-Tyr in mice treated with AZD3241 were comparable to the control group (**Figure 7**).

### AZD3241 Enhances Nrf2 and Haemoxygenase-1 (HO-1) Expression in the Colons of DSS Challenged Mice

**Figure 8:**
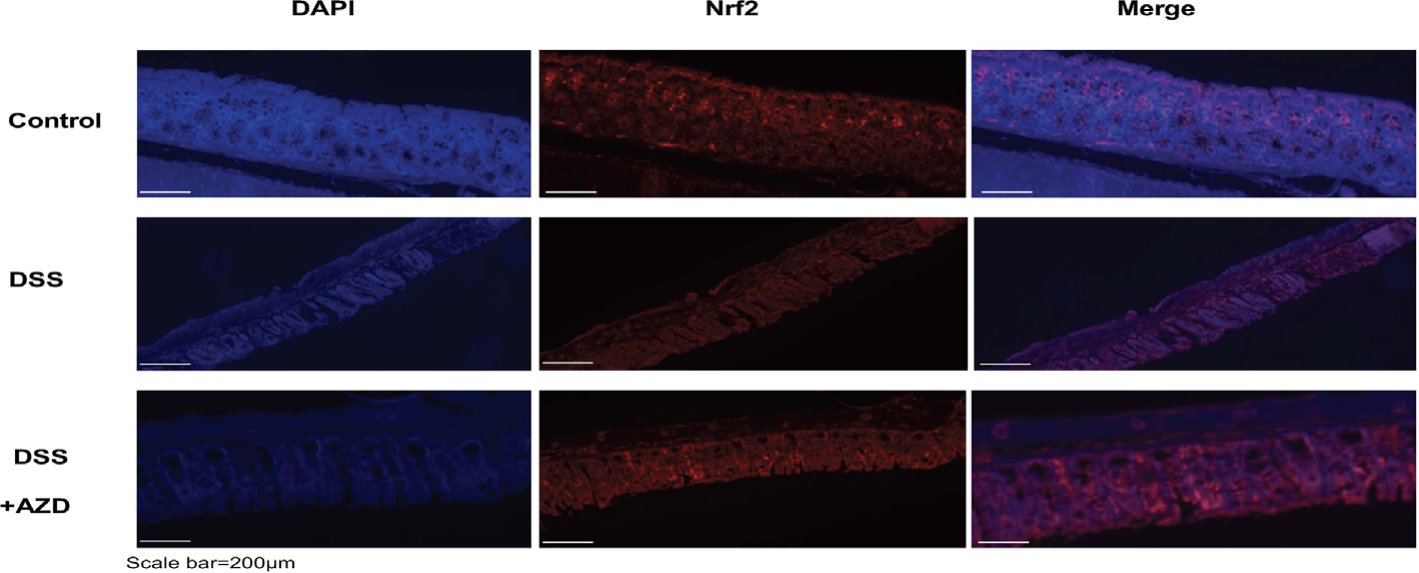
Representative Nrf2 immunofluorescence images of colon sections challenged with DSS for 9 days. Mice were randomly divided into three treatment arms over 9 days i) vehicle group (n = 5) given normal drinking water and peanut butter without drug; ii) dextran sodium sulfate (DSS) group (n = 5) given 2% w/v DSS in drinking water and peanut butter without drug; iii) DSS+AZD3241 (n = 5) given 2% w/v DSS in drinking water and AZD3241 @ 30 mg/kg in peanut butter. Colons were resected after 9 days of treatment, embedded in paraffin and sectioned for immunofluorescence studies as described in the methodology section. Representative images were taken from at least three fields of view from each colon at 20x magnification on a multifilter fluorescent microscope (ZEISS AXIOscope; Talavera Rd, Sydney, Australia). AZD3241 is abbreviated to AZD in this figure.

**Figure 9:**
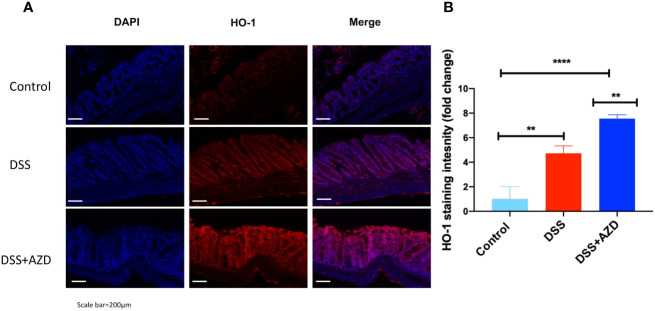
Representative HO-1 immunofluorescence images of colon sections challenged with dextran sodium sulfate (DSS) for 9 days in the absence or presence of AZD3241 supplementation. Mice were randomly divided into three treatment arms over 9 days i) vehicle group (n = 5) given normal drinking water and peanut butter without drug; ii) DSS group (n = 5) given 2% w/v DSS in drinking water and peanut butter without drug; iii) DSS+AZD3241 (n = 5) given 2% w/v DSS in drinking water and AZD3241 @ 30 mg/kg in peanut butter. **(A)** Colons were resected after 9 days of treatment, embedded in paraffin and sectioned for immunofluorescence studies as described in the methodology section. Representative qualitative images were taken from at least three fields of view from each colon at 20x magnification on a multifilter fluorescent microscope (ZEISS AXIOscope; Talavera Rd, Sydney, Australia). **(B)** Total fluorescent intensity was averaged across each series of images and presented fold change. Only three animals per group were imaged for these experiments. **p = 0.001, ****p < 0.001.

Expression of Nrf2 in experimental groups was assessed by immunofluorescence experiments on cut sections of colon tissue as described in the methods section. In mice insulted with DSS alone a marked decrease in the expression of Nrf2 was demonstrated compared to DSS+AZD3241 and control groups (**Figure 8**). Consistent with the activation of this transcription factor the level of expression for the downstream antioxidant response element HO-1 significantly higher in the DSS+AZD group compared to DSS alone (p=0.001) and control group control (p<0.001) (**Figure 9**). Collectively, these data indicate that AZD3241 stimulated the antioxidant function of Nrf2 (~2-fold above DSS insult alone), which manifested as an enhanced expression of HO-1 protein in accordance with the decreased in inflammation and oxidative tissue damage determined in the same mice.

## Discussion

Several anti-TNF drugs and more than 100 different antioxidants have been tested (Moura et al., 2015) as potential therapeutics for IBD but to date, none have translated successfully to a cure. One reason may be that the suite of therapeutics tested fail to target the central factor that underlies IBD pathogenesis. Neutrophil-MPO mediated inflammation and oxidative tissue damage may exacerbate colon inflammation. Thus, inhibiting MPO activity could potentially alleviate the inflammatory course of IBD. Herein, we demonstrate that orally available AZD3241 (a selective, irreversible MPO inhibitor) significantly ameliorates clinical manifestations of experimental IBD through inhibiting biochemical markers of colon damage typically raised during acute inflammation in this IBD model. We propose AZD3241 might offer therapeutic potential through mitigating MPO-HOCl production during colon inflammation, thereby preserving *in situ* antioxidant capacity and inhibiting oxidative tissue damage that contributes to the progression of this acute pathology.

Thus, AZD3241 significantly improves body weight and clinical score when compared to the absence of drug supplementation by inhibiting MPO-halogenating activity and oxidative damage that contribute to colon inflammation and suppressing neutrophil activation (judged by assessing calprotectin) (Chami et al., 2020). It is notable that neutrophils are the predominant cell type that are recruited early during acute inflammation and calprotectin represents ~60% of all soluble cytosolic proteins in these cells. Thus, calprotectin levels indirectly reflect neutrophil density in the acute inflammatory response (Bunn et al., 2001; Bjarnason, 2017). Hence, fecal calprotectin levels correlate positively with the neutrophil count in the inflamed mucosa of pediatric onset ulcerative colitis (Pakarinen et al., 2010) and is routinely used as a marker of neutrophil infiltration/activation in clinical IBD (Sipponen and Kolho, 2015). Therefore, although the drug-impact on fecal calprotectin did not reach statistical significance, any decrease in this biomarker is likely consistent with diminished colonic inflammation. Taking this into account, the simplest explanation is that the drug is active through inhibition of MPO halogenating ability rather than altering neutrophil recruitment to the inflamed colon (as demonstrated histopathological using the same tissues from mice co-supplemented DSS+AZD3241). Consistent with this notion, we demonstrated that colon homogenate 3-chloro-Tyr, a stable marker of MPO/HOCl mediated protein damage (Buss et al., 2003), was significantly lower in AZD3241-treated animals compared to mice insulted with DSS alone. Decreased concentration of 3-chlor-Tyr in the colon is a strong indicator of lower neutrophils/MPO activity in the tissue that manifests as decreased oxidative tissue damage, which is also reflected in the preservation of colon architecture shown in the AZD3241-treated animals. The biological efficacy of MPO inhibition by AZD3241 has been reported by others (Jucaite et al., 2015), however, this study is the first to investigate the impact of AZD3241 in an animal model of IBD.

Fecal hemoglobin, a surrogate measure for the presence of blood, is considered a reliable predictor of advanced colorectal neoplasia (Garcia et al., 2015; Navarro et al., 2019; Peng et al., 2019). Here we demonstrated that fecal Hb decreased significantly in mice supplemented AZD3241 vs mice treated with DSS alone, once again aligning with the concomitant preservation of colon surface epithelium and diminished rectal bleeding observed for the same mice. Live animal imaging based on oxidation of exogenous luminol also revealed significant attenuation in the bioluminescence signal detected specifically in the abdomen of DSS-treated mice. These data are completely consistent with the decreased level of the weak peroxidase hemoglobin and the active inhibition of MPO peroxidase both proteins capable of oxidizing luminol in the presence of hydrogen peroxide. Again, these improved biomarkers were linked to preservation of colon histology and overall improved clinical score for the same mice.

The extent of protection afforded to the gut by AZD3241, as determined using this bioluminescent approach, suggests that the drug inhibits total peroxidase activity. Luminol oxidation may be attributed to infiltrating MPO-containing neutrophils, accumulating hemoglobin, other endogenous tissue peroxidase enzymes and oxidants produced during acute inflammation. The observation that animals supplemented AZD3241 showed decreased fecal hemoglobin content while the extent of neutrophil recruitment to the inflamed bowel was not different between the mice insulted with DSS (with or without supplemented AZD3241) indicate that the loss in luminol bioluminescence determined here was not due to a major change in tissue MPO concentration. While it is feasible then to attribute the diminished signal to reduced bleeding in the gut, this outcome does not rule out a drug-mediated inhibition of MPO bioactivity in the inflamed intestinal wall as MPO is the preferred molecular target for AZD3241. A similar result has been demonstrated recently using the same luminol-based assay in mice (Chami et al., 2020) supplemented the MPO inhibitor 4-methoxy-tempo prior to insult with DSS (Rees et al., 2009). Likewise, 2-thioxanthines (compounds similar to AZD3241) are considered as a class of suicide inhibitors of MPO (Cheng et al., 2019) without impacting the function of other peroxidases (but still inhibits 90-98% extracellular MPO activity) thus MPO can continue its role in bactericidal activity (Tidén et al., 2011; Cheng et al., 2019). The current drug targets extracellular MPO, does not completely ablate MPO activity and is not taken up by cells so that intracellular MPO is unaffected and phagocytosis can continue hence defense against microorganisms is not completely inhibited. This is supported by our results showing unaltered recruitment of neutrophils in the colon which indicates that host immune response to chemotactic stimulus was effective in the drug-treated group. Furthermore, the drug is aimed at being administered acutely to reduce the impact of an IBD flare and so would not be given long term; this would not impact the host defense against infections.

Acute inflammation induced in this IBD model leads to oxidative stress which elicits inherent antioxidant tissue response through a variety of pathways. Of these, the Keap1-Nrf2 protein complex becomes activated under stress conditions to release the active transcription factor that stimulates a raft of antioxidant systems (Tu et al., 2019). The master regulator Nrf2 is known to mitigate intestinal inflammation (Yanaka, 2018) by downregulating NFκB, thereby inhibiting downstream proinflammatory cytokine release (Puscheck et al., 2015). Consistent with others, we have shown an upregulation of Nrf2 immunofluorescence in the AZD3241-treated group compared to mice treated with DSS alone, which showed a diminution of immunofluorescence signal for Nrf2. Of note, a lack of increased Nrf2 expression in colons from mice insulted with DSS alone is somewhat paradoxical considering that DSS challenge induced oxidative stress, which is anticipated to elicit the Nrf2 pathway. One possible explanation for this paradox could be the inhibitory effect of oxidative stress on Nrf2-mediated activation of antioxidant genes. Pathologically high levels of stress have shown to completely suppress the Nrf2 pathway (Fratta Pasini et al., 2012), which might be the case here where DSS challenge causes severe oxidative tissue damage. Similar results have been reported by others indicating lower Nfr2 expression levels in DSS alone treated group compared to vehicle group (Sangaraju et al., 2019). Thus, one plausible answer for this improvement could be that in addition to inhibiting MPO, AZD3241 activity may preserve the antioxidant capacity of Nrf2, leading to an increased expression of the protein that works in concert with MPO inhibition to maintain the histoarchitecture of the colon and minimize the extent of inflammation and bleeding into the colon lumen.

Another possible explanation for high Nrf2 activity in the AZD3241-treated group is an off-target impact that directly elicits higher levels of Nrf2 protein expression, thereby enhancing the colon tissue antioxidant response as indicated by higher haemoxygenase-1 (HO-1) expression levels which is transcriptionally regulated by Nrf2, and known to protect the colon against DSS induced intestinal inflammation (Li et al., 2018; Sangaraju et al., 2019). If this is the case, then it is expected that the tissue profile of antioxidant response elements will change and perhaps this may offer an alternate mechanism to protect the gut tissues through elevation of the antioxidant capacity in the bowel wall. For example, Nrf2 closely regulates cellular glutathione (GSH) levels by transcription of numerous reactive oxygen species (ROS)-detoxifying enzymes which are involved in the inactivation of ROS, thus minimizing oxidative stress-induced tissue/cellular damage (Tonelli et al., 2018). Similarly, Nrf2 is a key regulator of the thioredoxin based antioxidant response system required for the recycling of oxidized protein thiols (Stamler and Slivka, 1996). One limitation of the current study is that while we report global expression of Nrf2 in colon tissue, we have not investigated the nuclear component which is responsible for activation of antioxidant genes (Takagi et al., 2018). Nonetheless, elevated HO-1 expression as demonstrated here is an indication of stimulation of Nrf2 nuclear component. Overall, this alternate pathway of activity warrants further investigation to reveal the true mechanism of action of this drug *in vivo.*

In conclusion, our results reveal that orally available AZD3241 improves body weight, clinical outcomes of the disease and preserves the histoarchitecture of the colon, thus allowing absorption of essential nutrients from the intestine by minimizing the damage to surface epithelium. To the best of our knowledge, this positive outcome in an acute model of colitis is the first study to investigate the effect of AZD3241 using an experimental model of IBD. Further investigations using this drug in other chronic models of IBD, which more closely resembles the human inflammatory condition such as severe combined immunodeficiency mouse model (SCID) are warranted. Furthermore, the precise mechanism of action of this drug in murine models of IBD needs to be established. Thus, AZD3241 did not effectively inhibit the extent of neutrophil recruitment to sites of colon injury yet was effective in decreasing MPO-mediated tissue damage and the drug also elicited the expression of the transcription factor Nrf2 in the colon, which may ultimately play a role in the overall mechanism of protection exhibited by this drug as indicated by higher HO-1 expression. Importantly, an analog of AZD3241 that belong to the same class of mechanism-based thioxanthine inhibitors (Tidén et al., 2011) are protective in other inflammatory models, suggesting a potential for broader use of this class of drugs to ameliorate inflammatory disorders. For example, pharmacological inhibition of MPO with the analog AZM198 has been identified as a potential strategy to limit endothelial dysfunction (Cheng et al., 2019). Similarly, AZM198 afforded protection to the liver in a mouse model of obesity and hypertension (Piek et al., 2019). Thus, testing AZD3241 in chronic mouse model (SCID) which better recapitulate human IBD should be the next goal.

## Data Availability Statement

The raw data supporting the conclusions of this article will be made available by the authors, without undue reservation.

## Ethics Statement

The animal study was reviewed and approved by Animal Ethics Committee, University of Sydney.

## Author Contributions

Author contributions are summarized as follows: Contribution to the conceptualization of the study: PKW, GA, BC. Study methodology: BC, GA, YL, PSG, GF, XSW. Formal analysis and assessment of data: GA, BC, YL, GF, ALS, AG, PKW. Investigation: GA, BC, PKW. Data storage and curation: GA, BC, ALS, AG, PKW. Writing—original draft preparation: GA and PKW. Writing—Review and Editing draft versions of the manuscript: PKW, YL, PSG, GF, XSW. Primary supervision of the staff in the Redox Biology Group: PKW.

## Conflict of Interest

The authors declare that the research was conducted in the absence of any commercial or financial relationships that could be construed as a potential conflict of interest.
